# Comprehensive dose–response study of pulsed field ablation using a circular catheter compared with radiofrequency ablation for pulmonary vein isolation: A preclinical study

**DOI:** 10.1016/j.hroo.2023.09.005

**Published:** 2023-09-18

**Authors:** Jonathan C. Hsu, Rajesh S. Banker, Douglas N. Gibson, Tara Gomez, Dror Berman, Keshava Datta, Qi Chen, Shephal K. Doshi

**Affiliations:** ∗ Cardiac Electrophysiology Section, Division of Cardiology, Department of Medicine University of California San Diego, La Jolla, California; † Premier Cardiology, Newport Beach, California; ‡ Interventional Electrophysiology, Scripps Clinic and Prebys Cardiovascular Institute, La Jolla, California; § Biosense Webster, Irvine, California; ¶ Pacific Heart Institute, Santa Monica, California

**Keywords:** Catheter ablation, Pulmonary vein isolation, Pulsed field ablation, Irreversible electroporation, Preclinical model

**Figure:**
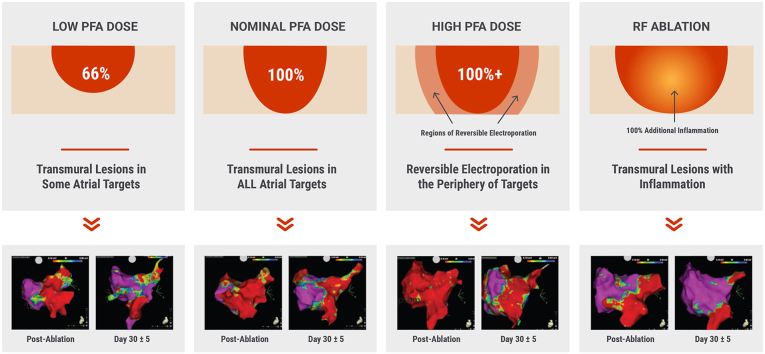

**Key Findings**

▪ In this preclinical study, we evaluated the efficacy and safety of low, nominal, and high doses of intracardiac pulsed-field ablation (PFA) compared with radiofrequency (RF) ablation in a porcine model and aimed to establish a dose-response relationship that could inform clinical study designs.
▪ At 30 days after ablation, complete pulmonary vein (PV) isolation was observed in 100% of PFA-treated and 83% of RF-treated animals.
▪ Histopathology showed that transmural lesions outside of the PV were observed less frequently in the low-dose group, while in the high-dose group regression in lesion volume on the posterior wall was seen over 30 days, indicating regeneration of ablated tissue at the periphery of the lesion.
▪ The nominal dose of PFA (12 applications per PV) resulted in transmurality throughout the atria and represents the optimal amount of PFA energy that provides permanent lesion placement and safety.

## Introduction

Pulsed field ablation (PFA) is a novel ablative method in which high-voltage, very-short-duration pulses create pores in the cytoplasmic membrane and cell death by a mechanism of irreversible electroporation.^1^ Different types of cells have different thresholds for the field strengths that induce cell death, with cardiomyocytes having one of the lowest threshold values.^2^ Thus, PFA has attracted interest as a potentially more selective method of cardiac ablation compared with radiofrequency (RF) energy, which could reduce the risk of collateral injury of neighboring structures while maintaining effectiveness. To date, evidence regarding a dose–response relationship for PFA in terms of effectiveness and safety is limited.

Previous preclinical studies using a proprietary PFA generator, novel circular catheter, and compatible mapping system, representing the first fully integrated mapping and PFA ablation system, showed that use of this PFA system for isolation of the pulmonary veins (PVs), superior vena cava, and right atrium was feasible, safe, and myocardial tissue–selective, producing durable lesions.^3^, ^4^, ^5^ The objectives of this study were to assess the efficacy and safety of various PFA doses delivered intracardially using the circular catheter compared with RF ablation in a porcine model and to establish a dose–response relationship that could inform future clinical study designs.

## Methods

In this study, 12 male Yorkshire pigs underwent ablation procedures using PFA, performed with the Biosense Webster (Irvine, CA) PFA Ablation System (TRUPULSE Generator and VARIPULSE Catheter) or RF, performed with a commercial system (THERMOCOOL SMARTTOUCH SF Catheter and SmartAblate Generator; Biosense Webster).

The animals were evaluated in 4 study groups: 3 groups received PFA treatment at low dose (1 application per location; 4 applications per PV), nominal dose (3 applications per location; 12 applications per PV), and high dose (6 applications per location; 24 applications per PV); and 1 control group received RF treatment (35 or 50 W, ≤60 seconds). All animals were survived for ≥28 days postablation to undergo follow-up electrophysiological procedures before being euthanized.

Gross necropsy was performed on all study subjects on day 30 ± 5, and histopathology was performed on all ablation sites. A licensed pathologist used a qualitative scoring system to rate the level of histopathologic findings, and the composite for all PFA dosage groups and all RF subjects was aggregated to compute an average rating.

During the study, the care and use of animals was conducted in accordance with the regulations of the US Department of Agriculture Animal Welfare Act. The research protocol was approved by the Institutional Animal Care and Use Committee and conformed to the Position of the American Heart Association on Research Animal Use. Full details of compliance with ethical guidelines, the study protocol, ablation procedures, pathology and histopathology assessments, and data analysis are provided in the Supplemental Methods.

## Results

### Safety

Phrenic nerve capture after pacing was successful in all animals, demonstrating no loss of phrenic function (Supplemental Figure 1). No clinically relevant reduction in PV diameter was noted for any animal immediately after ablation or on day 30 ± 5 follow-up procedures. Additional safety findings are summarized in the Supplemental Safety Results.

### Effectiveness

Elimination of PV potentials using a PENTARAY diagnostic mapping catheter (Biosense Webster) after the initial ablation procedure was confirmed in all animals, demonstrating 100% acute effectiveness in the left atrium.

**Figure 1:**
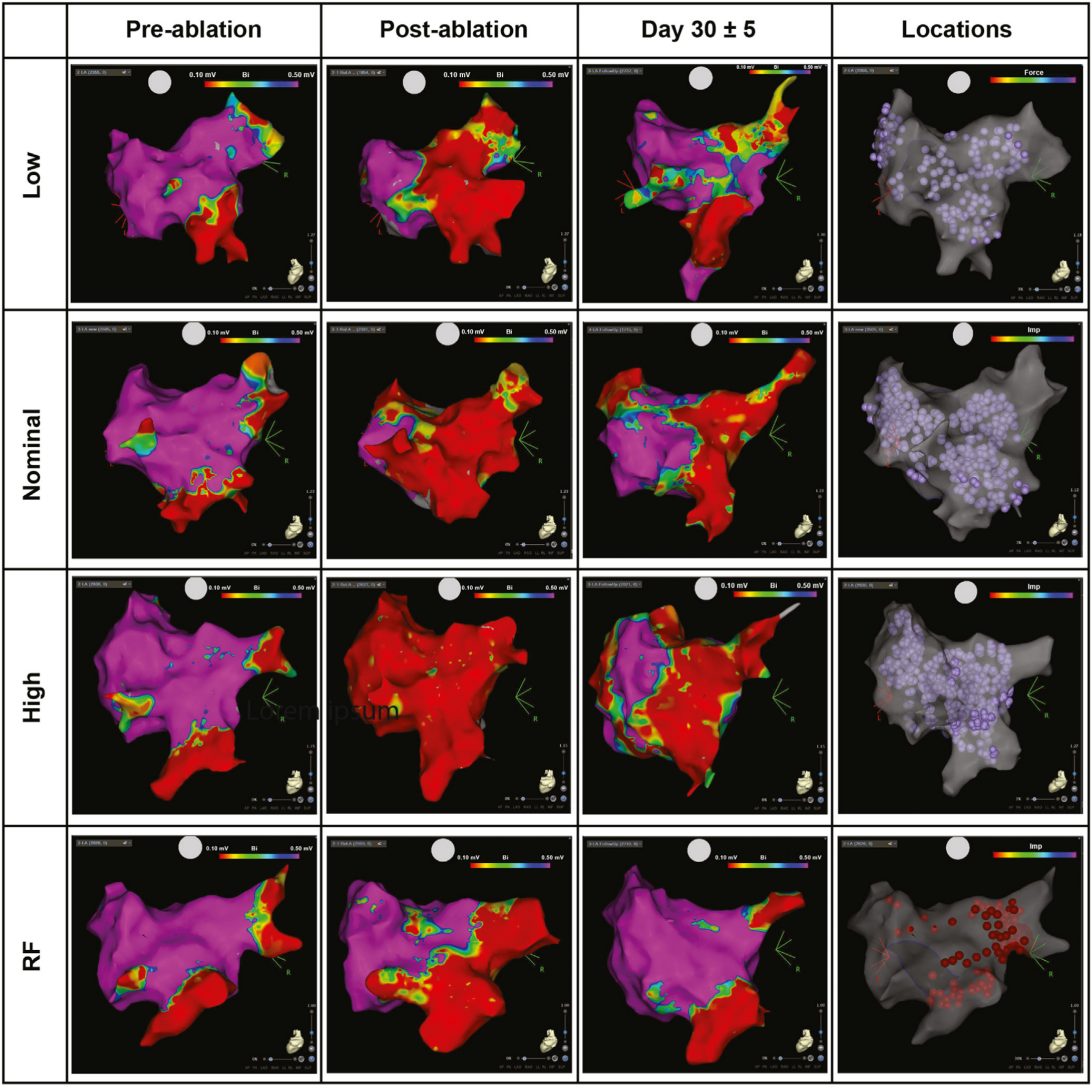
Voltage maps by study arm. Voltage maps and electrogram readouts preablation, postablation (acute), and after day 30 ± 5 (chronic). In ablation location images, *purple tags* indicate pulsed field ablation ablations and *red tags* indicate radiofrequency (RF) ablations. Voltage is represented on a color scale from *purple* (high voltage, healthy tissue) to *red* (low voltage, scar tissue).

On day 30 ± 5, complete (100%) chronic PV isolation was observed in all PFA-treated animals. In the RF group, 83.3% chronic PV isolation was achieved. Figure 1 shows representative voltage maps.

### Gross pathology

**Figure 2:**
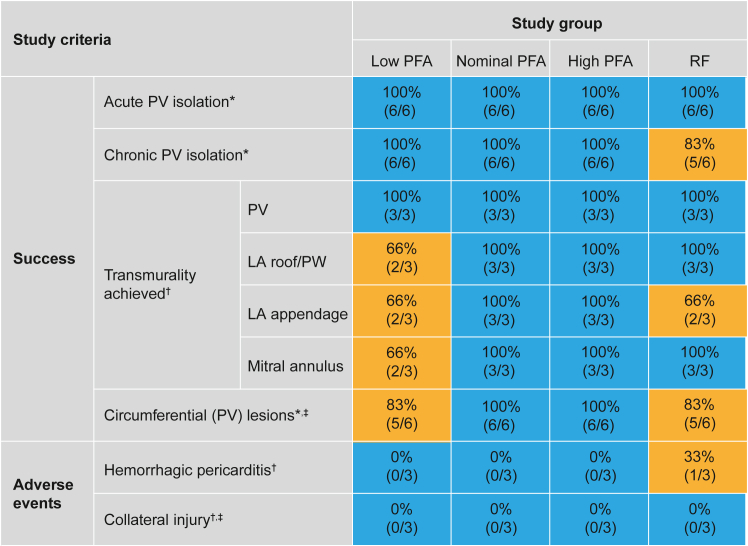
Summary of dose response. ∗N = 6 veins per group. †N = 3 animals per group. ‡Esophageal injury, phrenic nerve injury, and injury to upstream and downstream tissues. LA = left atrium; PFA = pulsed-field ablation; PV = pulmonary vein; PW = posterior wall; RF = radiofrequency.

There were no gross signs of collateral injury to anatomic structures adjacent to ablation sites, including the aorta, lungs, esophagus, pericardium, and phrenic nerve (Figure 2).

Based on gross and histopathologic findings, there were no significant differences among the 3 PFA doses. For RF-treated animals, treatment sites were within expectations for RF ablation. Lesions typically were transmural with pronounced fibrosis commensurate with the degree or severity of ablation. One animal in the RF group had unequivocal evidence of epicardial perforation leading to hemorrhagic pericarditis (Figure 2). Additional details are provided in the Supplemental Pathology Results.

### Histopathology

In PFA-treated animals, lesions at all target sites were transmural, with ablated cardiac tissue replaced by fibroplasia, or fibrosis present in amounts consistent with PFA lesions. The blood vascular network and nerve bundles seemed to have been preserved. No mineralization or necrosis was present, indicating normal healing progression. In RF-treated animals, myocardial necrosis often remained deep within ablated areas segregated by thick fibrous connective tissues.

**Figure 3:**
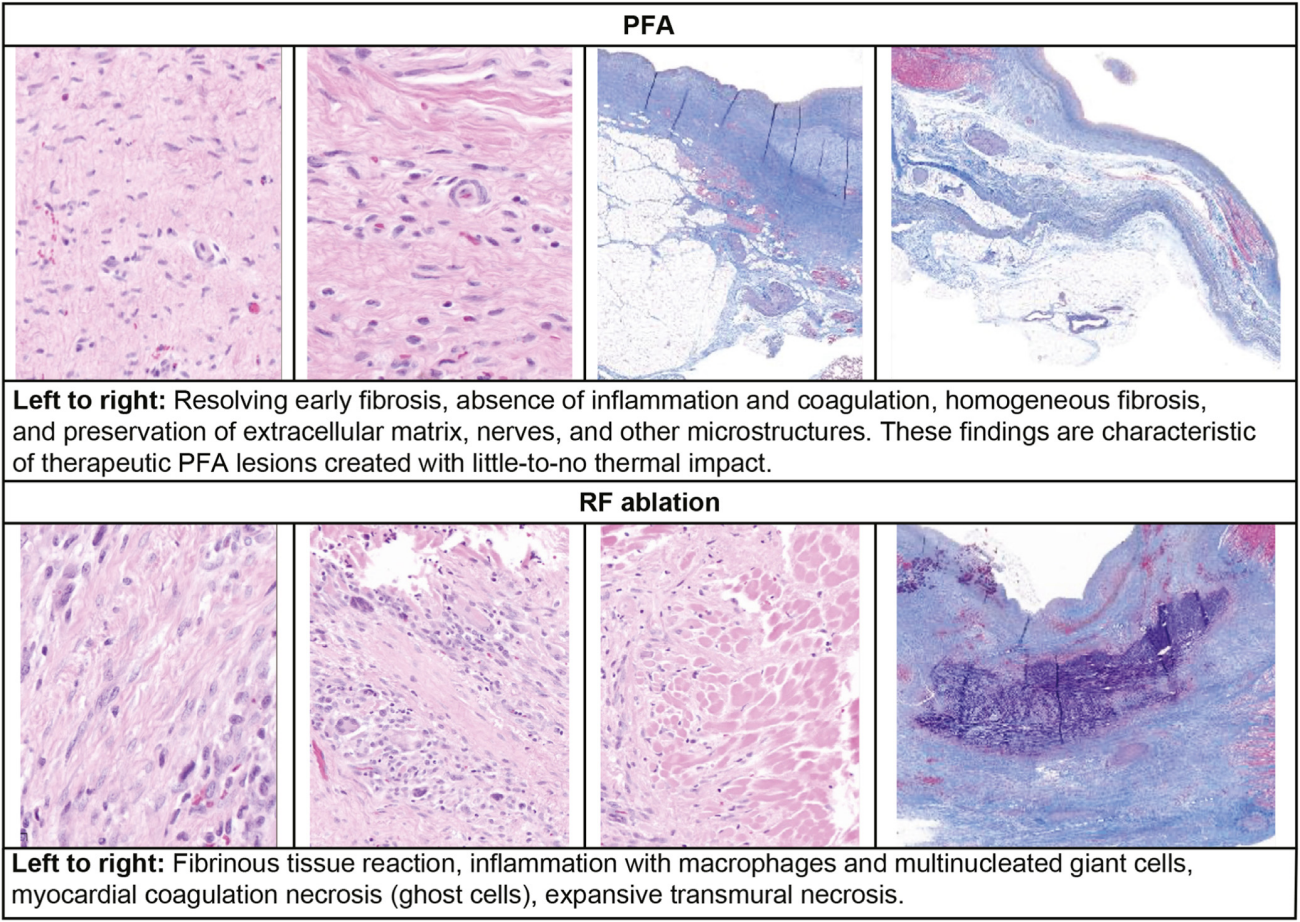
Histopathologic findings with pulsed field ablation (PFA) vs radiofrequency (RF) ablation.

**Figure 4:**
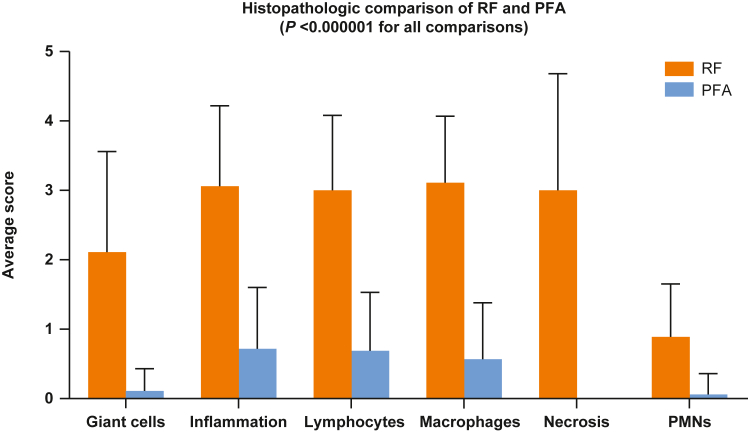
Histopathologic observations demonstrating large amounts of severe chronic inflammation in radiofrequency (RF)-treated tissue (based on average histopathologic score) in comparison to pulsed field ablation (PFA)–treated tissue with no or minimal inflammation. PMN = polymorphonuclear neutrophil.

All histopathologic findings were within expectations for their respective technologies. Both PFA- and RF-treated tissues produced the expected degree of postablation tissue modulation and repair, but the RF treatment arm showed severe chronic inflammation (Figures 3 and 4, and Supplemental Figure 2).

### Dose–response

All PFA-treated animals presented 100% acute and chronic PV isolation. However, per histopathology, transmural lesions in regions outside of the PV were observed less frequently in the low-dose group, which showed a similar pattern of effectiveness to the RF group.

### Voltage mapping in the high-dose group

The high-dose PFA group showed significant regression in ablation lesion volume on the posterior wall (PW) over 30 days, indicating reversible loss of electrical conduction of ablated tissue at the periphery of the lesion (Supplemental Figure 3).

## Discussion

Preclinical testing in a porcine, live beating heart model demonstrated that the novel fully integrated PFA system can safely create intracardiac lesions in a simulated clinical PV isolation procedure. The study further demonstrated a dose–response relationship with respect to the effectiveness of transmural and circumferential lesion formation. Nominal-dose PFA represents the workflow recommendations described in the inspIRE (Pulsed Field Ablation System for the Treatment of Paroxysmal Atrial Fibrillation by Irreversible Electroporation; ClinicalTrials.gov Identifier: NCT04524364) and admIRE (Assessment of Safety and Effectiveness in Treatment Management of Atrial Fibrillation With the BWI IRE Ablation System; ClinicalTrials.gov Identifier: NCT05293639) clinical studies. Dose–response analysis showed that PV isolation was achieved safely using the recommended nominal dose of 3 applications per location with 4 locations per PV, with a 2-fold margin of safety. These findings support those of previous preclinical studies in porcine models, which demonstrated the efficacy and safety of the PFA system for the creation of durable atrial lesions, even with subtherapeutic applications.^3^, ^4^, ^5^

These findings indicate that the PFA ablation system effectively produced transmural atrial myocardial lesions through irreversible electroporation without significant thermal contribution. In a side-by-side comparison of RF- or PFA-treated tissues, histopathologic and histologic outcomes were distinct. In PFA-treated animals, transmural lesions coincided with preservation of the myocardial microstructure and the absence of necrosis, in contrast to the coagulation necrosis and persistent inflammation seen in RF-treated animals, consistent with previous studies.^6^^,^^7^ Tabulated histologic observations demonstrated a significantly more severe immune response in RF-treated vs PFA-treated tissue (Figure 4), indicating the PFA-treated tissue had a distinct cell death mechanism that lacked the histologic hallmarks of thermal injury, the active mechanism in RF technology.^6^^,^^7^ In our study, the PW area that appears isolated by voltage map seems reduced at follow-up. This finding suggests that ablation at the periphery of the lesion is temporary. Similarly, in patients undergoing PV isolation with PFA, fused lesions on the PW and extensive lesion sets on the PW resulting in atrial tachycardia have been observed.^8^^,^^9^

### Study limitations

The limitations of our study are inherent to preclinical animal models with unknown correlations to humans, and, specifically in the context of PV ablation, some differences and variability in PV structures and branching may exist.

## Conclusion

Nominal doses of PFA resulted in transmurality throughout the atria, whereas low doses did not. Post–voltage map scar volumes were more similar than those created from high doses at chronic follow-up. In comparison to RF, all PFA doses showed a marked histologic distinction in chronic inflammation.
