# Supplementary material for: Comprehensive dose–response study of pulsed field ablation using a circular catheter compared with radiofrequency ablation for pulmonary vein isolation: A preclinical study
Source: Heart Rhythm O2. 2023 Sep 18;4(10):662–7. doi: 10.1016/j.hroo.2023.09.005 (PMC10626186; doi:10.1016/j.hroo.2023.09.005)
Supplement: Supplementary Material [file mmc1.docx]

**Supplementary Material**

**A Comprehensive Dose-response Study of Pulsed-field Ablation Using a Circular Catheter Compared With Radiofrequency Ablation for Pulmonary Vein Isolation: A Preclinical Study**

Jonathan C. Hsu, MD, MAS^a^; Rajesh S. Banker, MD^b^; Douglas N. Gibson, MD^c^; Tara Gomez, PhD^d^; Dror Berman, BSc^d^; Keshava Datta, PhD^d^; Qi Chen, PhD^d^; Shephal K. Doshi, MD^e^

^a^Cardiac Electrophysiology Section, Division of Cardiology, Department of Medicine University of California San Diego, La Jolla, CA, USA; ^b^Premier Cardiology, Newport Beach, CA, USA; ^c^Interventional Electrophysiology, Scripps Clinic and Prebys Cardiovascular Institute, La Jolla, CA, USA; ^d^Biosense Webster Inc., Irwindale, CA, USA; ^e^Pacific Heart Institute, Monica, CA, USA.

**Supplementary Methods**

The study included 12 healthy porcine subjects (male Yorkshire pigs) and was conducted at Absorption Systems (San Diego, CA, USA) in accordance with US Food and Drug Administration regulations on Good Laboratory Practice for Nonclinical Laboratory Studies. During the study, the care and use of animals was conducted in accordance with the regulations of the US Department of Agriculture Animal Welfare Act. The research protocol was approved by the Institutional Animal Care and Use Committee and conformed to the Position of the American Heart Association on Research Animal Use.

***Study Procedures***

Animals underwent ablation procedures under general anesthesia with mechanical ventilation. PFA ablation was performed with the Biosense Webster IRE Ablation System (TRUPULSE Generator and VARIPULSE Catheter), supported by the CARTO 3 System, nGEN irrigation pump and tubing, pacing system, and requisite cables. RF ablation was performed with an approved RF-based system (THERMOCOOL SMARTTOUCH SF Catheter and SmartAblate Generator; Biosense Webster) supported by appropriate accessory devices.

The live beating heart porcine model used in this study simulates a human-based clinical procedure that includes ≥1 PV as well as other anatomically relevant atrial locations (such as the left posterior wall) and high-risk areas (such as the mitral annulus and left atrial appendage).

A baseline intracardiac ultrasound (ICE) was performed to assess any preexisting abnormalities, such as effusion or mural thrombosis. The right atrium was mapped with a PENTARAY Nav Eco High Density Mapping Catheter (Biosense Webster). Pacing for the phrenic nerve on the posterolateral wall was performed and tagged, and the His bundle was located and tagged. The lateral free wall of the right atrium was ablated, after which the animal’s cardiac rhythm was recorded. A voltage re-map of the right atrium was performed with the PENTARAY catheter, the phrenic nerve was re-checked by pacing to confirm function, and a check for effusion was performed via ICE. Following standard catheterization techniques and trans-septal puncture, access to the left atrium was verified using ICE or via the fluoroscope. The left atrium was mapped with the PENTARAY catheter, then the ablation catheter was inserted into the left atrium. PV potential was recorded prior to PV ablation. Roof/posterior wall, mitral annulus, and appendage ablations were performed. After each set of ablations, the animal’s cardiac rhythm was recorded.

Twelve animals were evaluated in 4 study groups; 3 test groups that received PFA treatment at low-dose (1 application per location), nominal-dose (3 applications per location), and high-dose (6 applications per location), and 1 control group that received RF treatment (35 W or 50 W, up to 60 seconds; **Supplementary Table 1**). The terms ‘low’, ‘nominal’, and ‘high’ refer to the number of energy applications delivered before moving or rotating the catheter. Thus, low-dose entails 1 application before moving or rotating the catheter (ie, 1 x 4 = 4 applications per PV), nominal-dose entails 3 applications before moving or rotating the catheter (ie, 3 x 4 = 12 applications per PV), and high dose entails 6 applications before moving or rotating the catheter (ie, 6 x 4 = 24 applications per PV). Each PFA application included trains of biphasic pulses for a total application duration of approximately 250 milliseconds. A 10-second pause was implemented between each delivered application. A setting of 1800 V on the PFA generator for 10 electrodes was used. A constant irrigation flow rate of 4 mL/min was maintained during the procedure. All procedures were performed under anticoagulation with heparin and an activated clotting time range of 300-400 seconds. Each of 3 physician evaluators performed 1 procedure in each of the 4 study groups.

Anatomic target sites and chambers designated for assessing PFA and RF lesions were identified based on their relevance to the intended clinical procedure, including some considered higher risk (eg, atrial appendage and mitral annulus). Ablations were performed both in the right and left atrium using a clinically relevant workflow and device settings deemed by the participating physician to be appropriate for the anatomical location. Details of the ablations performed in the 4 study arms are shown in **Supplementary Table 1**. Acute PV isolation was assessed by demonstrating entrance block by pacing or, optionally, by checking for exit block. To assess the safety of ablation on phrenic nerve function, ablation energy was applied directly adjacent and endocardial to the phrenic nerve in all PFA study groups; in the RF group, energy was applied more conservatively adjacent and endocardial to the nerve (>5 mm in distance away from the nerve) so as not to risk the chronic survival of the animal. The location of the phrenic nerve was identified by performing pacing from a diagnostic catheter on the endocardial surface of the expected adjacent location of the phrenic nerve at least at 5 mA output, then marked on the map for later confirmation with ablation by similar pacing. Phrenic nerve stimulation was assessed by confirmation of contraction of the diaphragm utilizing visual and manual assessment of contraction, or by fluoroscopic assessment of visual contraction.

Baseline and post-ablation PV diameters were assessed by fluoroscopy and intracardiac ultrasound.

All animals were survived for ≥28 days post ablation to undergo follow-up electrophysiology procedures prior to being euthanized. During the follow-up electrophysiology procedures, a diagnostic catheter was inserted into the treated PV to check for electrical potential, and chronic PV isolation was assessed by checking for entrance block.

***Gross Pathology and Histopathology Assessments***

Gross necropsy was performed on all study subjects on Day 30 ± 5, consisting of a systematic examination of the animal’s general physical condition, as well as external and internal organs and tissues, focusing on assessment of cardiac trauma that could be related to the ablation procedure.

Histopathology was performed on all ablation sites. Paraffin blocks of target sites and any additional gross lesions in the heart were cut and stained with hematoxylin and eosin and elastin trichrome or equivalent. Peripheral/downstream tissues and noncardiac lesions were stained with hematoxylin and eosin only. Study slides were evaluated for localization, severity, and distribution of tissue injury.

Histopathologic examination of the treatment sites included evaluation for localization, severity, and distribution of tissue injury. Assessments included the presence of the following: giant cells, lymphocytes, macrophages and polymorphonuclear neutrophils, necrosis, and inflammation. A licensed pathologist used a qualitative scoring system to rate the level of findings:

0 = No finding

1 = Minimal

2 = Mild

3 = Marked

4 = Severe

The composite for all PFA dosage groups and all RF subjects was aggregated to compute an average qualitative rating.

***Data Analysis***

In-life performance ratings were summarized in tabular format, and calculations of means and standard deviations were calculated as applicable.

**Supplementary Results**

***Safety Results***

Throughout the procedure, no signs of respiratory distress, pain, or other abnormal behaviors were observed in any of the study animals. Additionally, no mural thrombus, coagulum or charring, cardiac effusion, or tamponade were noted for the duration of the study; there also was no evidence of steam pop detected in any of the treated animals.

No clinically relevant reduction in PV diameter was noted for any animal immediately following ablation or on Day 30 ± 5 follow-up (remap) procedures. The greatest acute and chronic reductions in PV diameter were seen in a single RF-treated animal (11.71% at the right superior PV and 22.93% at the right inferior PV, respectively). However, across all animals, there was no average acute or chronic reduction in diameter of either treated PV.

***Pathology***

Fibrinous pericarditis of undetermined cause was observed in 2 animals, 1 in the nominal-dose PFA group and 1 in the RF group. Fibrinous pericarditis is not uncommon in the swine model and does not necessarily point to a specific cause, although subclinical pericarditis due to infectious agents was considered highly likely. A second animal in the RF group had hemorrhagic pericarditis accompanied by unequivocal evidence of epicardial perforation leading to hemorrhage into the pericardial sac; this ablation was performed using the parameters described for RF ablation in **Supplementary Table 1**. Seven RF applications in the right inferior pulmonary vein and one application in the right superior pulmonary vein at 35 W were not completed. Such injuries are rare but may occur during cardiac catheter insertion and manipulation in the heart chambers.

**Supplementary Table 1.** Ablation parameters and locations.

| **Treatment** | **Target anatomic region** | | **Active electrodes^†^** | **Applications** | | **Post-application delay (sec)** |
| --- | --- | --- | --- | --- | --- | --- |
|  |  |  |  | **In series (dose)** | **Per structure** |  |
| Low-dose PFA | LA | PV (≥1) | 10 | 1 | 4 | 10 |
|  |  | Roof/posterior wall | 10 | 1 | 4 | 10 |
|  |  | Appendage | 10 | 1 | 4 | 10 |
|  |  | Mitral annulus | 6 | 1 | 1 | 10 |
|  | RA | Lateral wall | 6 | 1 | 4 | 10 |
| Nominal-dose PFA | LA | PV (≥1) | 10 | 3 | 12 | 10 |
|  |  | Roof/posterior wall | 10 | 3 | 12 | 10 |
|  |  | Appendage | 10 | 3 | 12 | 10 |
|  |  | Mitral annulus | 6 | 3 | 3 | 10 |
|  | RA | Lateral wall | 6 | 3 | 12 | 10 |
| High-dose PFA | LA | PV (≥1) | 10 | 6 | 24 | 10 |
|  |  |  | 10 | 6 | 24 | 10 |
|  |  | Roof/posterior wall | 10 | 6 | 24 | 10 |
|  |  | Appendage | 10 | 6 | 24 | 10 |
|  |  | Mitral annulus | 6 | 6 | 6 | 10 |
|  | RA | Lateral wall | 6 | 6 | 24 | 10 |
| RF ablation^‡^ | **Target anatomic region** | | **Target power (W)** | **Cut-off temp (°C)** | **Duration (sec)** | **N (minimum)** |
|  | LA | PV (≥1) | 35 | 40 | 30-60 | 10 |
|  |  | Roof/posterior wall | 35 | 40 | 60 | 4 |
|  |  | Appendage | 35 | 40 | 60 | 4 |
|  |  | Mitral annulus | 50 | 40 | ≤30 | 1 |
|  | RA | Lateral wall | 50 | 40 | 60 (drag) | 1 |
| ^†^Deactivation of up to 4 electrodes allowed in cases of overlap.  ^‡^Target contact force = 5-30 g; irrigation 15 mL/min. | | | | | | |

LA = left atrium; PFA = pulsed-field ablation; PV = pulmonary vein; RA = right atrium; RF = radiofrequency.

**Supplementary Figure 1.** Phrenic nerve challenge. Phrenic nerve functionality challenged via direct application of ablation energy (representative image from each arm). Purple tags indicate PFA ablations, red tags indicate RF ablations, and yellow tags indicate the location of the phrenic nerve. Post-procedure phrenic nerve functionality was 100% in all animals.

^
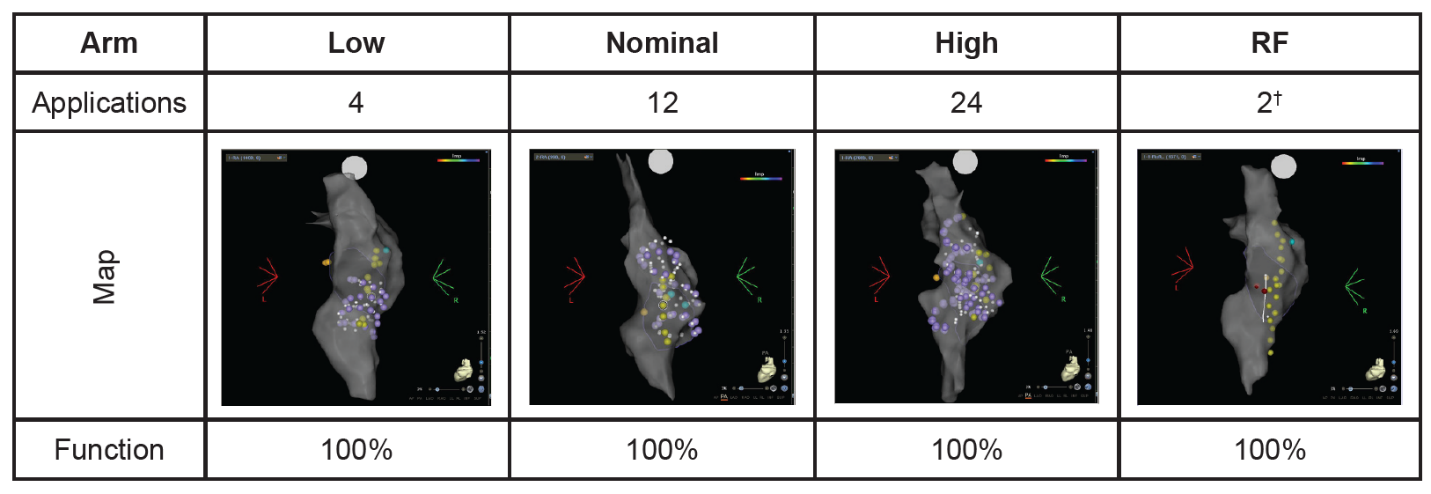
^

^†^RF applied more conservatively, adjacent to the nerve to avoid jeopardizing chronic survival.

PFA = pulsed field ablation; RF = radiofrequency.

**Supplementary Figure 2.** Histopathologic characteristics of thermal lesions in cardiac ablation.


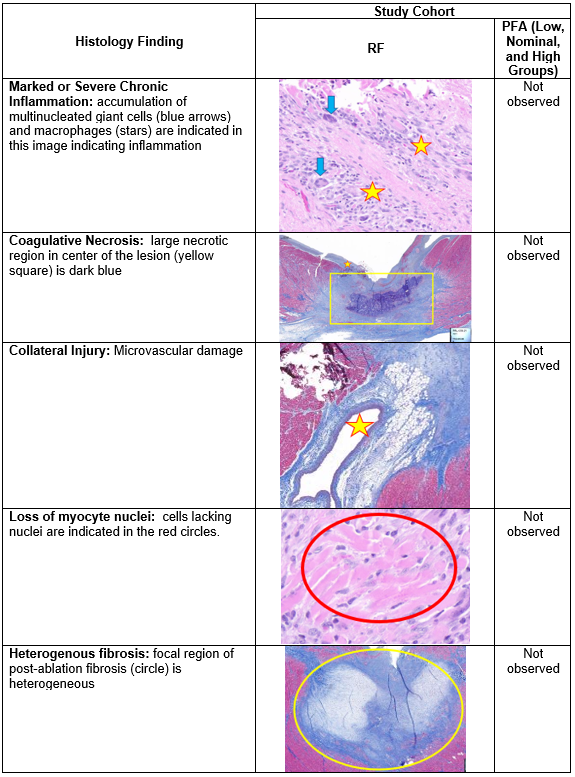


PFA = pulsed field ablation; RF = radiofrequency.

Supplementary Figure 3. Voltage maps from the high-dose PFA group pre-ablation, post-ablation (acute), and after Day 30 ± 5 (chronic). In ablation location images, purple tags indicate PFA ablations, red tags indicate RF ablations, and yellow tags indicate the location of the phrenic nerve. Note, voltage is represented on a color scale of purple (high voltage, healthy tissue) to red (low voltage, scar tissue).


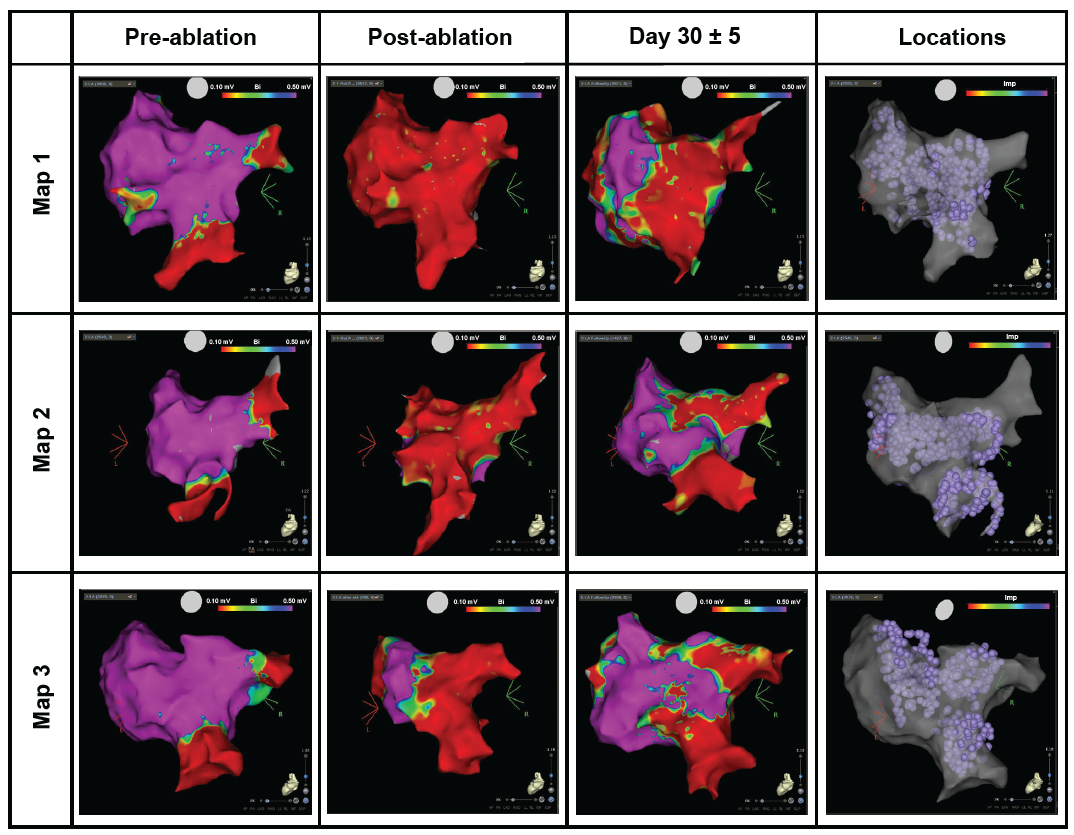


PFA = pulsed field ablation; RF = radiofrequency.
